# Changes to the upper gastrointestinal microbiotas of children with reflux oesophagitis and oesophageal metaplasia

**DOI:** 10.1099/mgen.0.000870

**Published:** 2022-09-15

**Authors:** Laurence D. W. Luu, Harveen Singh, Natalia Castaño-Rodríguez, Steven T. Leach, Stephen M. Riordan, Nicodemus Tedla, Usha Krishnan, Nadeem O. Kaakoush

**Affiliations:** ^1^​ School of Medical Sciences, UNSW Sydney, NSW 2052, Australia; ^2^​ School of Biotechnology and Biomolecular Sciences, UNSW Sydney, NSW 2052, Australia; ^3^​ Department of Pediatric Gastroenterology, Sydney Children’s Hospital, Randwick, NSW 2031, Australia; ^4^​ School of Women’s and Children’s Health, UNSW Sydney, NSW 2052, Australia; ^5^​ Gastrointestinal and Liver Unit, The Prince of Wales Hospital, Randwick, NSW 2031, Australia

**Keywords:** oesophagus, metaplasia, microbiota, paediatric, *Prevotella*

## Abstract

Little is known of the relationships among paediatric upper gastrointestinal microbiotas, and the impact of medication use and disease on their diversity. Here, we investigated the diversity of three microbiotas in the upper gastrointestinal tract of paediatric patients in relation to each other and to host factors. Oral, oesophageal and gastric microbiotas from a prospective paediatric cohort (*n*=54) were profiled using the 16S rRNA gene and ITS2 amplicon sequencing. 16S rRNA gene amplicon sequencing of oesophageal biopsies from a retrospective paediatric cohort (*n*=96) and shotgun metagenomics data from oesophageal brushings (*n*=88) were employed for genomic signature validation. Bacterial diversity and composition showed substantial differences across oral, oesophageal and gastric fluid samples that were not replicated for fungi, and the presence of reflux led to increased homogeneity in the bacterial component of these three microbiotas. The oral and oesophageal microbiotas were associated with age, sex, history of oesophageal atresia and presence of oesophageal metaplasia, with the latter characterized by *Prevotella* enrichment. Proton pump inhibitor use was associated with increased oral bacterial richness in the gastric fluid, and this correlated with increased levels of gastric pro-inflammatory cytokines. Profiling of oesophageal biopsies from a retrospective paediatric cohort confirmed an increased *Prevotella* prevalence in samples with metaplasia. Analysis of metagenome-derived oesophageal *Prevotella melaninogenica* genomes identified strain-specific features that were significantly increased in prevalence in samples with metaplasia. *Prevotella* enrichment is a signature associated with paediatric oesophageal metaplasia, and proton pump inhibitor use substantially alters the paediatric gastric microenvironment.

## Data Summary

16S rRNA gene and ITS2 amplicon sequencing data sets for the prospective and retrospective cohorts were generated in this paper and have been deposited in the European Nucleotide Archive (PRJEB48716) and can be accessed using the following link: https://www.ebi.ac.uk/ena/browser/view/PRJEB48716. The shotgun metagenomics data set from oesophageal brushings was previously deposited in the European Nucleotide Archive (PRJEB25422) and can be accessed using the following link: https://www.ebi.ac.uk/ena/browser/view/PRJEB25422.

**Impact Statement**

Our study profiled the upper gastrointestinal microbiomes of paediatric patients, identifying age, reflux, medication use and metaplasia to be variably associated with these microbiotas. Specific genomic features within *Prevotella melaninogenica* were identified to be enriched in esophageal metaplasia.

## Introduction

The oesophageal microbial community remains an understudied microbiome that has increasingly been associated with disease [1–3]. While a substantial portion of oesophageal bacteria can be tracked to the oral cavity, the human oesophageal microbiota in adults is a distinct community from that within the saliva [4]. Whether this is also the case in children is yet to be determined, and elucidating this would provide insights into the development of the oesophageal microbiota.

To date, evidence exists showing that the oesophageal microbiota is altered in gastro-oesophageal reflux disease (GERD), Barrett’s oesophagus (BE), oesophageal adenocarcinoma (EAC), oesophageal squamous cell carcinoma and eosinophilic oesophagitis (EE) [1–3, 5]. However, most research has been performed in adults where variables such as alcohol intake, smoking and obesity can act as confounders. Several studies have examined the paediatric oesophageal microbiota, with an emphasis on examining differences in diversity between controls and patients with EE. The oral and oesophageal microbiotas of 33 paediatric patients with EE and 35 controls were compared, and an enrichment of *Proteobacteria* in EE was found [6]. In both children and adults, increased bacterial load in EE and GERD was observed when compared to controls, as well as an increase in *Haemophilus* in untreated patients with EE and a decrease in *Streptococcus* in patients with GERD [7]. Further, differences between the oesophageal microbiotas of patients with EE and controls were identified, with patients treated with swallowed topical corticosteroids having a microbial shift towards the controls [8].

Oesophageal atresia (EA) is a congenital malformation of the oesophagus, often with a fistulous communication to the trachea that is repaired in the neonatal period. These children have oesophageal dysmotility and significant risk of GERD and the subsequent replacement of the squamous epithelial lining with a metaplastic columnar epithelium (metaplasia) [9], and thus represent a naïve population to examine microbiota changes in disease. To our knowledge, no study has holistically investigated the microbiotas in the upper gastrointestinal tract of paediatric patients with a history of EA or at the time of metaplastic transformation.

Here, we profiled the bacterial and fungal components of the upper gastrointestinal microbiomes of prospectively recruited paediatric patients. We identified age, reflux and medication use as variably influencing these microbiotas, while *Prevotella* was associated with oesophageal metaplasia. We confirmed increased prevalence of *Prevotella* in oesophageal metaplasia in a retrospective paediatric cohort, identifying genetic features within *Prevotella melaninogenica* strains enriched in metaplasia by performing comparative analyses on genomes originating from shotgun metagenomes.

## Methods

### Prospective cohort

Paediatric patients were prospectively recruited at Sydney Children’s Hospital in 2018 (Table 1). Exclusion criteria were any antibiotic use in the preceding 2 months. Fifty-four patients undergoing routine surveillance were recruited, of which four were sampled a second time 6–9 months after the first collection. Oral swabs (total *n*=58) and oesophageal biopsies (total *n*=58) were obtained, while gastric fluid samples were not collected during nine visits (total *n*=49). Samples were collected while patients were under general anaesthetic and frozen at −80 °C until use. The tracheal aspirate microbiota was previously profiled in a subset of these patients [10]. The mean age was 7.8 years (0.6–18 years; sd: 5.2) and 26/54 (48.1 %) were female. Thirty of 54 (55.6 %) patients had a history of EA. GERD, defined as reflux oesophagitis on biopsy and/or an abnormal result on pH/impedance testing, was present in 21/54 (38.9 %). EE was defined as >15 eosinophils per high-power field, in addition to clinical symptoms of oesophageal dysfunction, with GERD excluded on the basis of normal pH impedance testing at the time of endoscopy, which sampled the proximal, middle and distal oesophagus. EE was seen in 13/54 (24.1 %). Metaplasia was defined as the replacement of any portion of the normal distal squamous epithelial lining by metaplastic columnar epithelium that was visible endoscopically ≥1 cm above the gastro-oesophageal junction and confirmed by histopathology [11]. Metaplasia was detected in 6/54 (11.1 %), and this was either gastric- (5/6; 83.3 %) or intestinal-like (1/6; 16.7 %). Thirty-seven of 54 (68.5 %) of children were on proton pump inhibitors (PPIs) and 13/54 (24.1 %) were on swallowed topical steroids for EE. See Table S1 (available in the online version of this article) for extended metadata.

**Table 1.** Clinical characteristics of the paediatric cohort prospectively recruited to the study. Participants were stratified according to history of oesophageal atresia

	EA (*n*=30)	No EA (*n*=24)
Female (%)	16 (53.3)	10 (41.7)
Age* (years)	7.2±5.2	8.4±5.2
Histology† (%)		
Normal	10 (33.3)	9 (37.5)
RE	14 (46.7)	7 (29.2)
EE	5 (16.7)	8 (33.3)
Fungal hyphae	1 (3.3)	0 (0)
Metaplasia‡ (%)	6 (20.0)	0 (0)
PPI use (%)	22 (73.3)	15 (62.5)
Topical steroid use (%)	7 (23.3)	6 (25.0)

*Mean age ±standard deviation of patients on first recruitment to the study.

†Results on histopathological assessment of oesophageal biopsy.

‡Detection of gastric or intestinal metaplasia.

EA, esophageal atresia; EE, eosinophilic oesophagitis; PPI, proton pump inhibitors; RE, reflux oesophagitis.

### Validation cohort

Formalin-fixed parafilm-embedded (FFPE) oesophageal tissue samples (*n*=96) previously collected (2015–2017) from paediatric patients undergoing endoscopy for evaluation of their symptoms were obtained from South Eastern Area Laboratory Services (SEALS) Pathology Laboratories. The mean age was 6.5 years (0.3–17.8 years; sd: 5.0) and 48/96 (50.0 %) were female. GERD was detected in 65/96 (67.7  %) of the patients, 12 of whom had an additional diagnosis of EA and one had a further diagnosis of short gut. Other clinical indications included EA with no GERD (*n*=27), constipation (*n*=1), abdominal pain (*n*=1), short gut (*n*=1) and no indication (*n*=1).

### FFPE sample preparation

Samples were prepared for DNA extraction and histology at the Garvan Institute of Medical Research Histopathology facility (Sydney, Australia). FFPE samples were either sectioned at 14 µm for DNA extraction or 4 µm and stained with haematoxylin and eosin for histopathological assessment by a blinded observer.

### Nucleic acid extraction

DNA was extracted from oesophageal biopsies, oral swabs and gastric fluid using the Allprep PowerViral DNA/RNA kit (Qiagen; Chadstone, VIC, Australia). DNA was extracted from oesophageal FFPE tissue using the AllPrep DNA/RNA FFPE kit (Qiagen).

### 16S rRNA gene and ITS region amplicon sequencing and analysis

The v4 region of the 16S rRNA gene and the fungal ITS2 region were profiled using Illumina MiSeq 2×250 bp chemistry as previously described [10, 12]. The ITS2 region was selected as it has previously been shown to be a suitable discriminatory marker when the full ITS region is not available and does not face the same human DNA amplification issues as the 18S rRNA gene [13]. Raw 16S rRNA gene reads were analysed using Mothur v1.39.1 and v1.42.3 [14], and the MiSeq standard operating procedures with some minor modifications (e.g. maxhomop=15, chimera.vsearch) [15]. Raw ITS2 reads were screened by quality, not aligned, clustered at 5 % by abundance. The taxonomic references used were RDP v16 for the 16S rRNA gene and UNITE v6 for ITS2. Raw reads ±sd for the 16S rRNA gene sequencing were 62 655±31 803 reads for the prospective cohort and 86 324±50 491 reads for the validation cohort. Read depths for 16S rRNA gene data and ITS2 data from the prospective cohort were 46 481 and 34 415 average clean reads/sample, respectively (Tables S2, S3), with the total number of OTUs being 6709 and 11 227. Rarefaction curves showed sequencing saturation, enabling comparisons of alpha diversity measures across sample types (Fig. S1a). Two negative (mock sampling in the clinic) controls were included in the experimental procedures for the prospective cohort (Fig. S1b–d). Operational taxonomic units (OTUs) that were robustly detected in the negative controls (see Tables S2 and S3) were removed from the count tables prior to the statistical analyses. The read depth for the 16S rRNA gene data for the validation cohort was 8907 clean subsampled reads/sample (Tables S4 and S5). The total numbers of OTUs prior to and following subsampling were 4948 and 2972, respectively. Six negative controls (buffers) and two parafilm section controls were included in the analysis. Despite the negative controls showing substantial separation from the oesophageal samples (Fig. S1e, f), the oesophageal FFPE samples were considered to be heavily contaminated due to the taxonomic classification of the OTUs (Table S4).

### Multiplex cytokine and chemokine assay

Thirty-four cytokines and chemokines were measured in the gastric fluid samples using the Cytokine and Chemokine 34-Plex Human ProcartaPlex Panel 1A (Jomar Life Research; Scoresby, VIC, Australia) as previously described [10].

### Statistical analysis

Alpha diversity measures (Margalef’s species richness, Pielou’s species evenness and Shannon’s diversity index) were calculated using Primer-E v6. For comparisons across location (oral, oesophageal and gastric), Kruskal–Wallis (KW) tests were employed as data were not normally distributed (Shapiro–Wilk test). Adjusted *P*-values were then calculated using Dunn’s multiple comparisons test. These tests were performed using GraphPad Prism v9. The *P*-value was calculated using a distance-based linear model (selection criterion: *R* ^2^, permutations=999) on Euclidean distances that included all clinical variables (patient, presence of EA, age, sex, PPI use, topical steroid use, histology results and detection of metaplasia) applied in Primer-E v6.

Beta diversity was interrogated by first calculating Bray–Curtis dissimilarities from OTU relative abundances (%) that were transformed by square root. Principal coordinate analysis (PCoA), analysis of similarities (ANOSIM; one way with 999 permutations), distance-based linear models (DistLM; selection criterion: *R* ^2^, permutations=999) and constrained ordination using distance-based redundancy analysis (dbRDA; maximum number of axes=10) were then calculated using Primer-E v6. Clinical predictors tested using DistLM and dbRDA were patient, age, sex, PPI use, topical steroid use, history of EA, diagnosis on endoscopic screening and presence of metaplasia.

To establish how distant the three upper gastrointestinal microbiotas were from each other intra-patient, average distances from centroid of the oral, oesophageal and gastric microbiotas within each patient were calculated using the PERMDISP function in Primer v6. Distances were then tested against the clinical variables listed above. Variables that were found to be significant were assessed using linear regression and Pearson’s correlation (age) and using analysis of variance (ANOVA) with a Dunnett’s multiple comparisons test (histology) within GraphPad Prism v9.

Per taxon analyses were performed using LEfSe [16]. This was performed through a two-step process. First, strict settings of all-vs-all and LDA effect size >2 were set to calculate true LDA effect sizes. Second, all cut-offs were removed to generate a list of *P*-values that was corrected for false discovery rate (FDR) using the practical Benjamini–Hochberg procedure. Only taxa that had an LDA effect size >3, *P*<0.05 and *q*<0.2 were considered. The change in prevalence of *Prevotella* and *Prevotella* OTU36 across subject groups stratified by histology was tested using the chi-square for trend test in GraphPad Prism v9.

Source tracking of microbial taxa (i.e. calculating the contribution of a source to a sink) was performed using SourceTracker [17]. Differences in the contributions of the oral and gastric microbiotas to the oesophageal microbiota were tested using a Kruskal–Wallis test with a Dunn’s multiple comparison test using GraphPad Prism v9 as data were not normally distributed (Shapiro–Wilk test). Differences in the contribution of the oral and oesophageal microbiotas to the gastric microbiota in the context of PPI use (yes/no) were tested using an unpaired *t*-test as data were normally distributed (Shapiro–Wilk test).

The association between gastric bacterial species richness and the gastric inflammatory profile [Euclidean distances of log(*x*+1)-transformed abundance of the 34 molecules] was tested using DistLM (selection criterion: *R* ^*2*^, permutations=999). Correlations between individual cytokines or chemokines and gastric bacterial species richness were tested using Pearson’s correlations in GraphPad Prism v9, and *P*-values were corrected for FDR using the practical Benjamini–Hochberg procedure. Associations between individual cytokines or chemokines [log(*x*+1)-transformed abundances] and gastric microbiota composition (Bray–Curtis resemblance from square root-transformed bacterial OTU relative abundances) were tested using DistLM (selection criterion: *R* ^2^, permutations=999).

### Analysis of genomic features from shotgun metagenomics data

Data comprising shotgun metagenomic sequencing of oesophageal samples from an adult cohort of GERD (*n*=30) and metaplasia (*n*=5, intestinal; *n*=6, gastric) as well as normal oesophagi (*n*=47) were previously published by our group [18]. The READ_QC module from MetaWRAP v1.4 [19] was used for read trimming and removal of human reads. Reads were mapped using PanPhlAn3 v3.1 and the *P. melaninogenica* pangenome [20]. The *P. melaninogenica* pangenome was downloaded using the function panphlan_download_pangenome.py -i Prevotella_melaninogenica. To establish the presence or absence of *P. melaninogenica* genes within samples, the lenient setting option (--min_coverage 1 --left_max 1.70 --right_min 0.30) was used (Table S6). To identify differential features, Fisher’s exact tests were applied on feature prevalence between normal and MET samples using the R package stats v4.1.3 (fisher.test). Next, chi-square for trend tests were applied on features found to be significantly different on Fisher’s exact using the R package rstatix v0.7.0 (prop_trend_test) and *P*-values were corrected for FDR using the Benjamini–Hochberg method. Hierarchical clustering analysis (HCA) was performed using Morpheus by Broad Institute (RRID:SCR_017386). Protein domain analyses of gene signatures were performed using InterPro [21].

## Results

### The paediatric oesophageal microbiota was compositionally distinct but mostly originates from the oral cavity

**Fig. 1.**
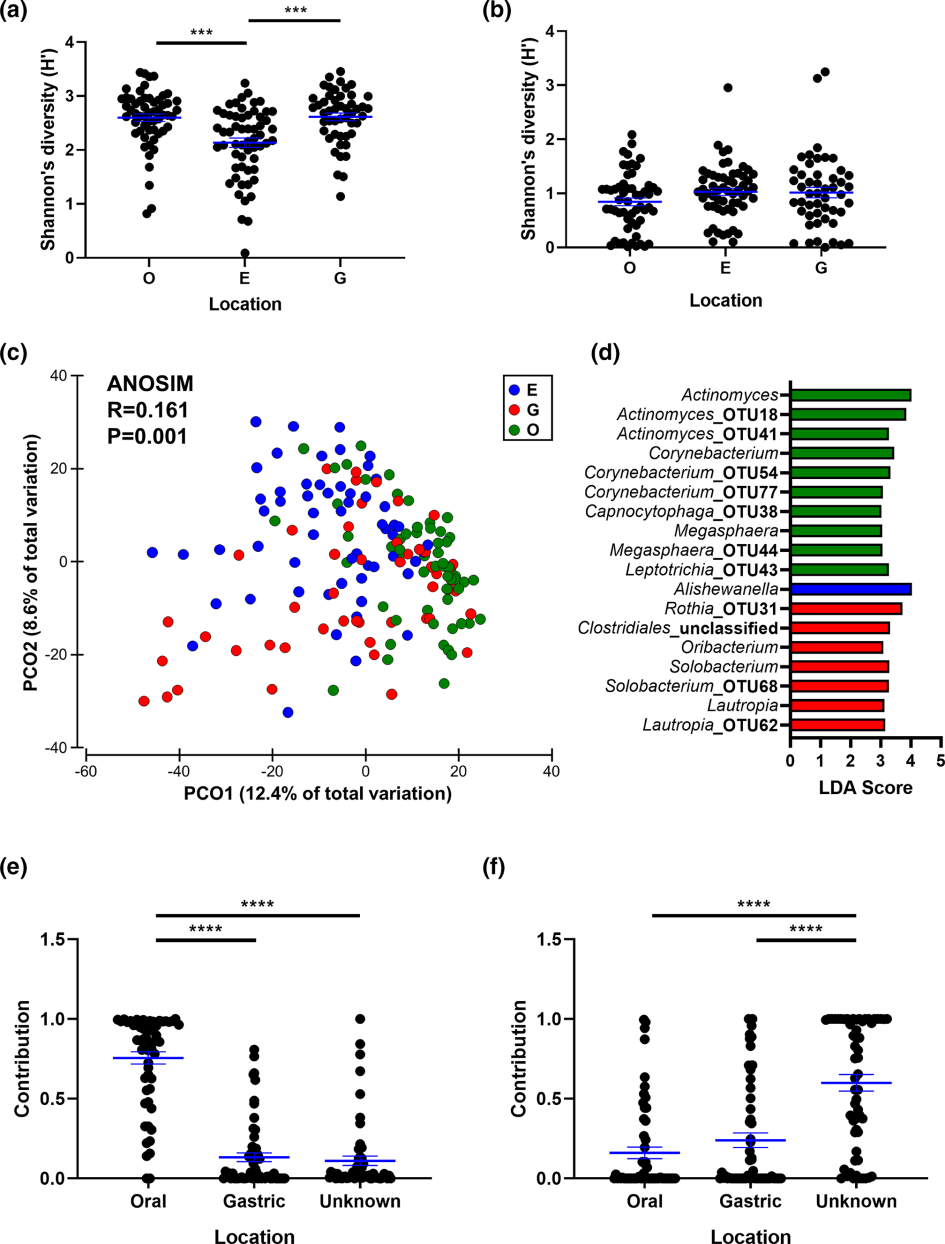
Upper gastrointestinal tract microbiota diversity in a prospective paediatric cohort. (a) Bacterial Shannon’s diversity across oral (**O**), oesophageal (**E**) and gastric fluid (**G**) samples. Data from one oral sample were considered an outlier and removed as they were found to have >3400 bacterial OTUs. Main test: H=22.83, *P*<0.0001, *n*=164. (b) Fungal Shannon’s diversity across oral, oesophageal and gastric fluid samples. Main test: H=3.9, *P*=0.14, *n*=162. Diversity measures were compared using a Kruskal–Wallis test with a Dunn’s multiple comparison test. (c) Principal coordinate analysis of bacterial beta diversity across sample type. The ordination was generated from a Bray–Curtis resemblance matrix on square root-transformed bacterial relative abundances. ANOSIM was employed to test for significant differences in beta diversity. (d) Bacterial taxa identified to be differentially abundant across the three sample types using LEfSe. Only genera and OTUs with an LDA score >3 were selected, all of which had *q*<0.05. (e) Contribution of oral and gastric fluid samples to oesophageal bacterial composition. (f) Contribution of oral and gastric fluid samples to oesophageal fungal composition. Contributions were calculated using SourceTracker and compared using a Kruskal–Wallis test with a Dunn’s multiple comparison test. ***P<0.001, ****P<0.0001.

Matched oral swabs, oesophageal biopsies and gastric fluid samples were collected from paediatric patients (Table 1), and the bacterial and fungal components of the microbiota were profiled. Alpha diversity was lowest within the oesophagus. Bacterial species richness (Dunn’s adjusted *P*<0.0001, post-hoc test following KW), species evenness (Dunn’s adjusted *P*=0.041, post-hoc test following KW) and Shannon’s diversity (Dunn’s adjusted *P*=0.0001, post-hoc test following KW) differed between oral and oesophageal samples, while bacterial species evenness (Dunn’s adjusted *P*=0.0013, post-hoc test following KW) and Shannon’s diversity (Dunn’s adjusted *P*=0.0001, post-hoc test following KW) were lower in oesophageal as compared to gastric fluid samples (Fig. 1a, Fig. S2a). In contrast, only fungal species richness was different between oral and oesophageal samples (Dunn’s adjusted *P*=0.0008, post-hoc test following KW), and this was higher in oesophageal samples (Fig. 1b, Fig. S2b).

Bacterial beta diversity was significantly different among all locations (pairwise *R* values=0.118–0.207, *P*=0.001 for all; ANOSIM) (Fig. 1c). Fungal beta diversity was also significantly different among locations (Fig. S2c); however, the effect size was small, driven by a difference between oral and oesophageal samples (*R*=0.07, *P*=0.001, ANOSIM), with other pairwise comparisons not being significant. Bacterial taxa that were differentially abundant among locations were identified (Fig. 1d). Eighteen genera and OTUs were found to be discriminatory features (LDA score >3, *q*<0.05) enriched in either oral, gastric fluid or oesophageal samples.

The contributions of the oral and gastric fluid microbiotas to the oesophageal microbiota were then calculated using source tracking. Despite the significant differences in bacterial beta diversity, most oesophageal bacteria were source tracked to the oral cavity and not the gastric environment (Fig. 1e). This was not observed for oesophageal fungi, the majority of which were of unknown origin (Fig. 1f).

### Age and reflux were associated with distances of the upper gastrointestinal microbiotas from each other intra-patient

**Fig. 2.**
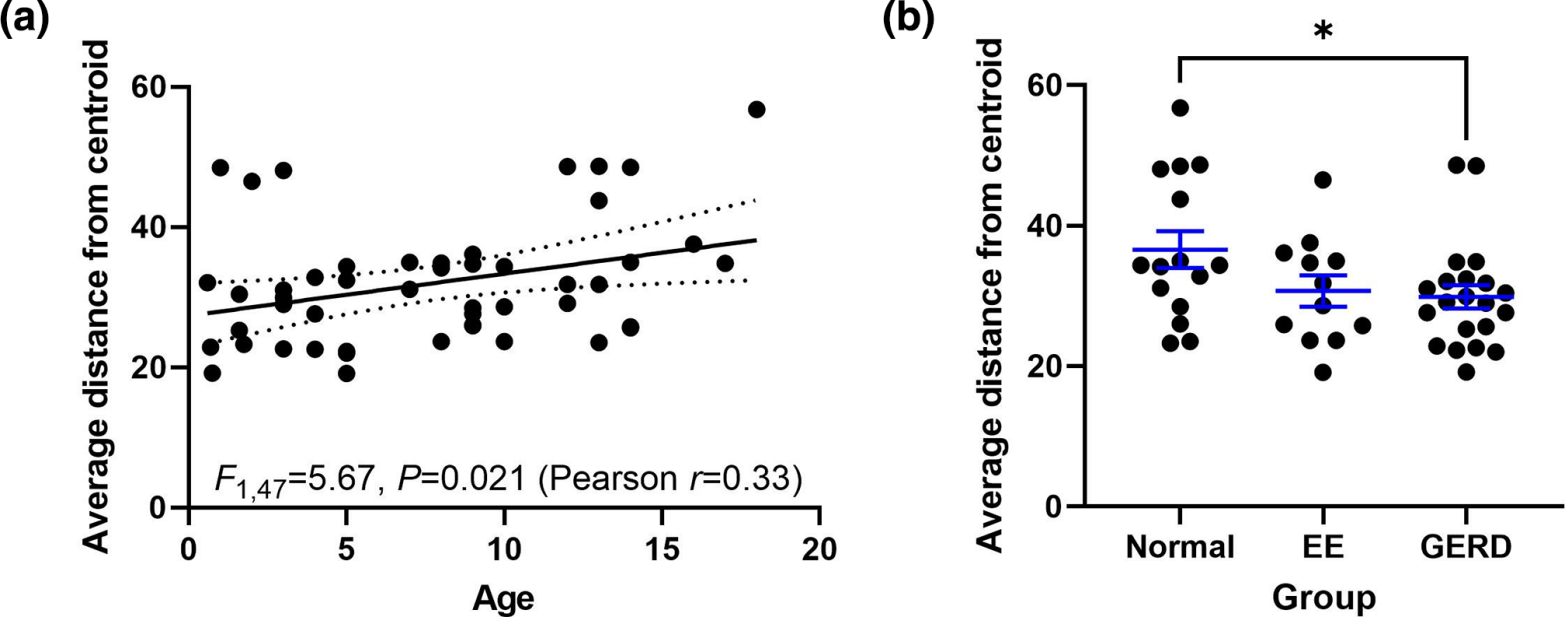
Relationship of intra-patient average distance from the centroid of the upper gastrointestinal tract microbiotas with host factors. (a) Relationship between age and distance from centroid. Distances from centroid of the three microbiotas within each patient were calculated and the relationship was tested using linear regression and Pearson’s correlation. (b) Distance from centroid in subjects stratified by results of histology. EE, eosinophilic oesophagitis; GERD, gastro-oesophageal reflux disease. Groups were compared using one-way ANOVA with a Dunnett’s multiple comparisons test.

The intra-patient average distance from centroid of the three microbiotas was calculated (i.e. how distant their compositions are from each other within each patient), limiting the analysis to patients with all three samples. The impact of clinical variables (patient, presence of EA, age, sex, PPI use, topical steroid use, histology results and detection of metaplasia) were then assessed. Age (pseudo-*F*=5.7, *P*=0.026, df=47; DistLM) and histology results (pseudo-*F*=4.7, *P*=0.014, df=47; DistLM) were associated with the intra-patient average distance from the centroid. Age was found to be positively correlated with the distance (Fig. 2a), while GERD (average distance: 29.9±1.7, *P*=0.045; ANOVA with post-hoc Dunnett’s) but not EE (average distance: 30.7±2.2, *P*=0.14; ANOVA with post-hoc Dunnett’s) was associated with significantly lower distances when compared to patients with normal oesophagi (average distance: 36.6±2.6) (Fig. 2b). In support, while the three microbiotas remained significantly different from each other inter-patient in each of the patient groups (normal: *R*=0.19, *P*=0.001; GERD: *R*=0.13, *P*=0.001; EE: *R*=0.22, *P*=0.001), the ANOSIM R statistic was lower in the patients with GERD.

### Metaplasia in the esophagus is associated with differences in oral and oesophageal microbiotas

**Fig. 3.**
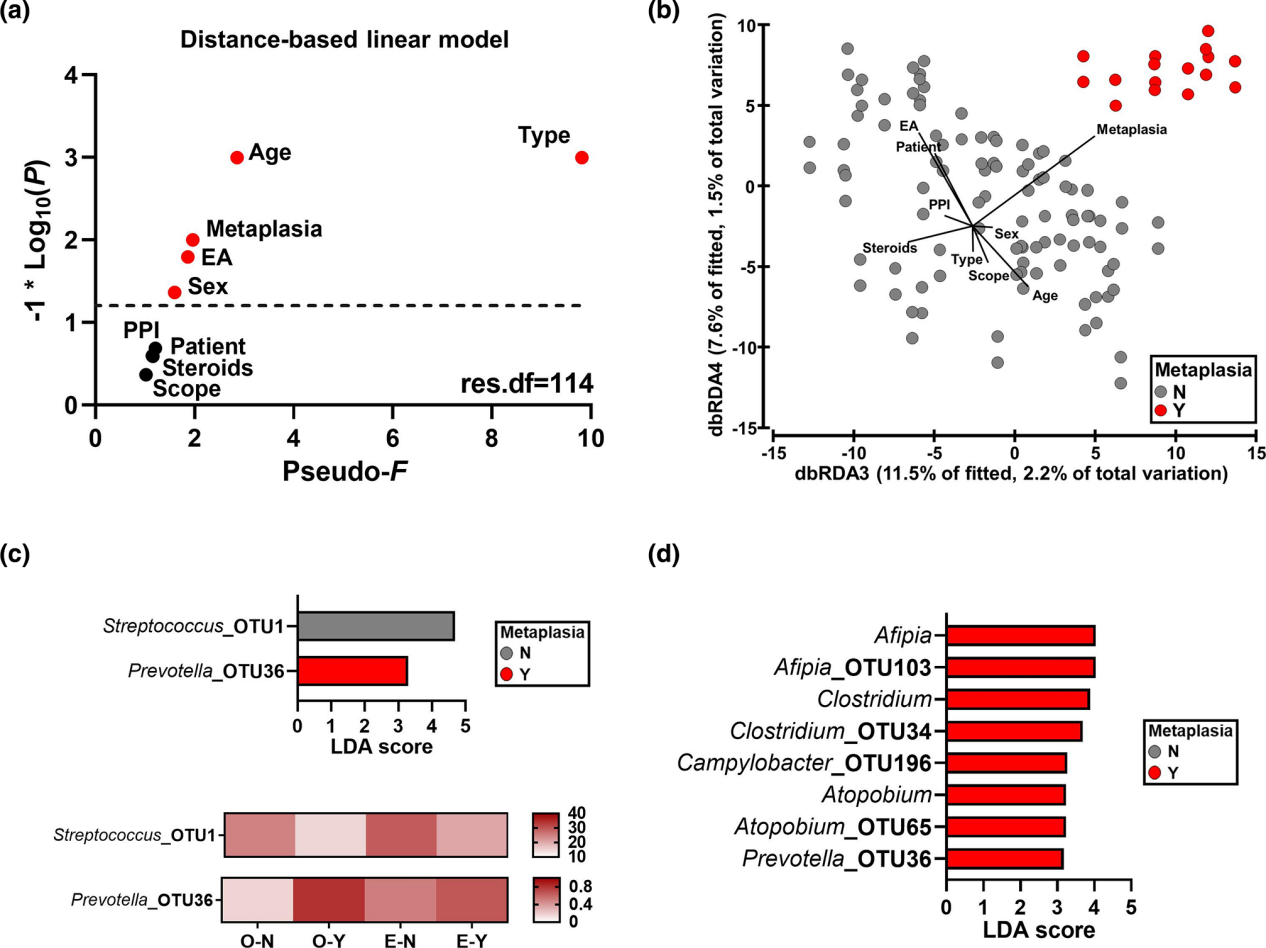
Association of oral and oesophageal microbiotas with clinical variables. (a) Statistics generated from a distance-based linear model examining the effects of clinical variables on the oral and oesophageal microbiotas. Points in red were found to be significantly associated with bacterial composition. EA, oesophageal atresia; PPI, proton pump inhibitor; scope, histology results. (b) Constrained ordination of axes 3 and 4 showing differences between presence (**Y**) and absence (**N**) of metaplasia. Constrained ordination was performed using distance-based redundancy analysis (dbRDA) applied on a Bray–Curtis resemblance matrix from square root-transformed bacterial relative abundances. (c) Bacterial taxa identified to be differentially abundant between the presence and absence of metaplasia using LEfSe (above) and their mean relative abundances within groups (below). Sample type (O or E) was selected as a sub-class and only taxa with an LDA score >3, *P*<0.05 and *q*<0.2 were reported. (d) Oesophageal bacterial taxa identified to be significantly differentially abundant between the presence and absence of metaplasia using LEfSe. Analysis was restricted to oesophageal samples and only taxa within the top 200 OTUs and with an LDA score >3, *P*<0.05 and *q*<0.2 were reported. *Afipia* was originally classified as *Bradyrhizobiaceae* unclassified and was adjusted on blastn search.

Given the compositional relationship between the oral and oesophageal microbiotas as well as the lack of relationship between the oesophageal and gastric microbiotas, as revealed by source tracking, we first examined the association of clinical variables [patient, type (i.e. location), presence of EA, age, sex, PPI use, topical steroid use, histology results and detection of metaplasia] with differences within the oral and oesophageal microbiotas together. Type of sample (oral vs oesophageal) was the only variable significantly associated with bacterial richness (pseudo-*F*=144.9, *P*=0.001, df=113; DistLM) and Shannon’s diversity (pseudo-*F*=17.3, *P*=0.002, df=113). While type of sample was also the strongest correlate of beta diversity differences, showing the highest test statistic, age, presence of metaplasia, history of EA and sex were also significantly associated with microbiota composition (Fig. 3a). This was confirmed by performing a constrained ordination using distance-based redundancy analysis on Bray–Curtis resemblances, where the effect of type of sample was observed on axis 1 (Fig. S3a, b), the effect of age and, to a lesser extent, history of EA on axis 2, and the effect of metaplasia on axis 3 (Fig. 3b, Fig. S3a). This revealed that differences observed in metaplasia were distinct from those observed with age and EA. Since metaplasia was only present in patients with a history of EA, the DistLM analysis was also repeated excluding non-EA patients, and metaplasia remained a significant predictor of beta diversity (pseudo-*F*=1.8, *P*=0.016, df=64).

Differences associated with metaplasia were then explored at the bacterial taxon level using LEfSe. Decreases in the dominant *Streptococcus* OTU1 (99.21 % similarity to *S. infantis *, *S. peroris *, *S. rubneri *, *S. oralis* and *S. australis *; LDA score=4.68, *P*=0.018, *q*=0.17) and increases in *Prevotella* OTU36 (99.21 % similarity to *P. melaninogenica *; LDA score=3.30, *P*=0.000046, *q*=0.0013) were observed in both oral and oesophageal samples (Fig. 3c). This difference was confirmed when oral and oesophageal samples were analysed separately (oral: LDA score=3.54, *P*=0.0035, *q*=0.019; oesophageal: LDA score=3.18, *P*=0.0072, *q*=0.16; Fig. 3d). *Afipia *, *Clostridium* and *Campylobacter* were also enriched (LDA score >3) in the metaplastic oesophagus (Fig. 3d).

### PPIs and gastric inflammation are associated with an altered gastric fluid microbiota

**Fig. 4.**
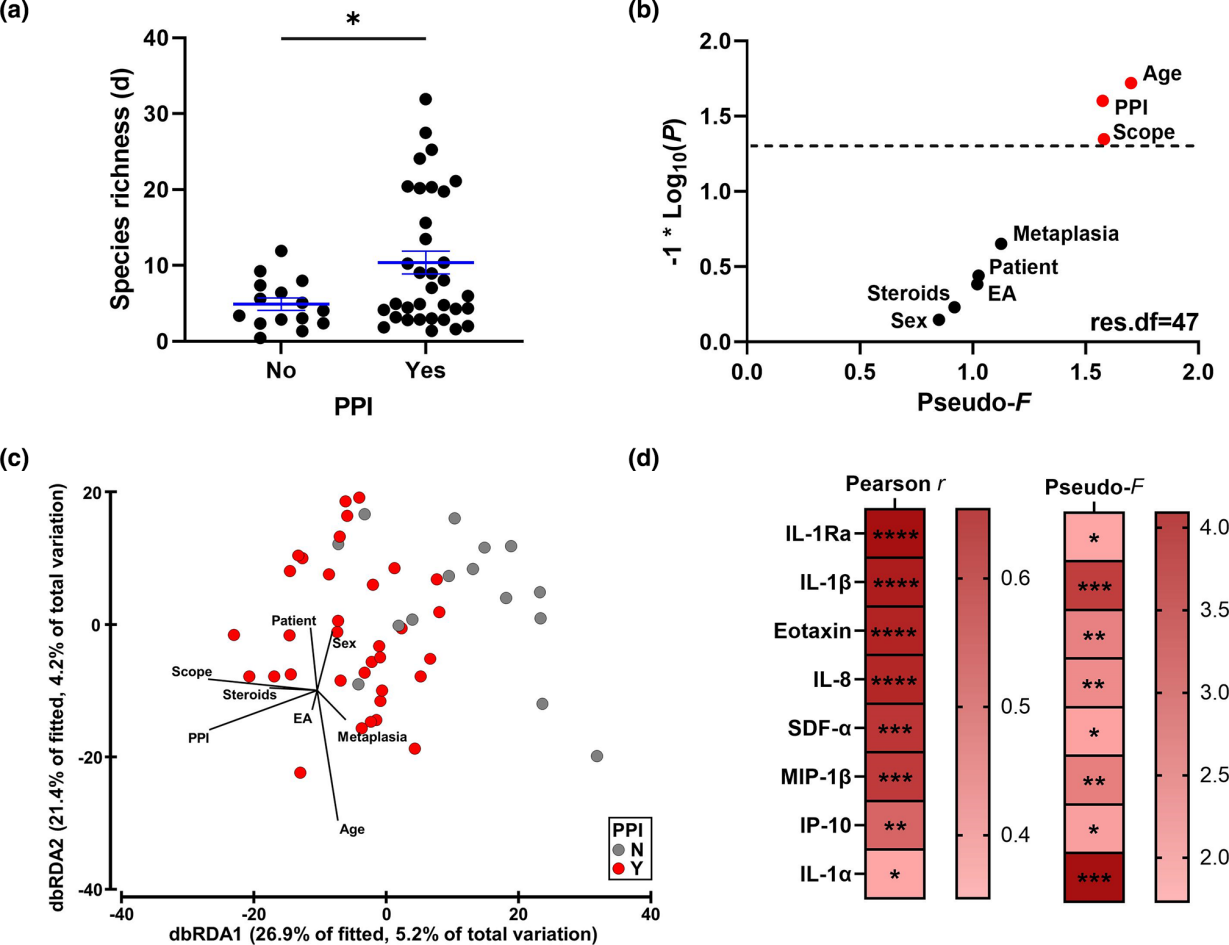
Association of gastric fluid microbiota with clinical variables. (a) Margalef’s species richness in proton pump inhibitor use. The *P*-value was calculated using a distance-based linear model on Euclidean distances correcting for all other variables. (b) Statistics generated from a distance-based linear model (Bray–Curtis resemblance matrix) examining the effects of clinical variables on the gastric fluid microbiota. Points in red were found to be significantly associated with bacterial composition. EA, oesophageal atresia; PPI, proton pump inhibitor; scope, histology results. (c) Constrained ordination of axes 1 and 2 showing differences in PPI use (**Y**) or not (**N**). Constrained ordination was performed using distance-based redundancy analysis (dbRDA) applied on a Bray–Curtis resemblance matrix from square root-transformed bacterial relative abundances. (d) Association of individual cytokines or chemokines with species richness (Pearson *r*, FDR) or bacterial composition (pseudo-*F*, DistLM). Only molecules associated with both measures were reported. Gradients refer to the values of the Pearson *r* and pseudo-*F*, respectively. *P<0.05, **P<0.01, ***P<0.001, ****P<0.0001.

Association of the same clinical variables with the gastric fluid microbiota was assessed. PPI use was the only variable for bacterial richness (pseudo-*F*=5.4, *P*=0.026, df=47, DistLM), with an increase in bacterial species richness observed in patients on PPIs (Fig. 4a). Significant associations with age, PPI use and histology results were observed for beta diversity (Fig. 4b), but not for detection of oesophageal metaplasia. Constrained ordination suggested that PPI use and histology assessment were explained on axis 1 and age on axis 2 (Fig. 4c, Fig. S4). To examine these changes further, the contributions of the oral and oesophageal bacteria to the gastric fluid bacteria were assessed using source tracking in the context of PPIs. A significant increase in the contribution of the oral microbiota with PPI use was identified (no: 26.7±5.8 % vs yes: 49.2±5.4 %, *P*=0.016; unpaired *t*-test) and this was at the expense of contributions from unknown sources (no: 44.6±9.9 % vs yes: 23.7±5.1 %, *P*=0.045; unpaired *t*-test) but not oesophageal microbiota contribution (no: 28.7±8.0 % vs yes: 27.1±4.7 %, *P*=0.85; unpaired *t*-test).

To determine whether gastric microbial changes were associated with inflammation, 34 cytokines and chemokines were measured. Gastric bacterial species richness was a strong predictor of cytokine and chemokine production (pseudo-*F*=19.95, *P*=0.001, df=45; DistLM). To reveal the cytokines and chemokines associated with bacterial changes, molecules correlated with bacterial richness (Pearson, FDR-corrected) and those associated with gastric microbiome composition (Bray–Curtis, DistLM) were identified (Fig. 4d). Eight molecules, including three involved in IL-1 signalling, were significantly associated with bacterial signatures (Fig. 4d).

### Increased prevalence of *Prevotella* in oesophageal biopsies with metaplasia was observed in a validation cohort

**Fig. 5.**
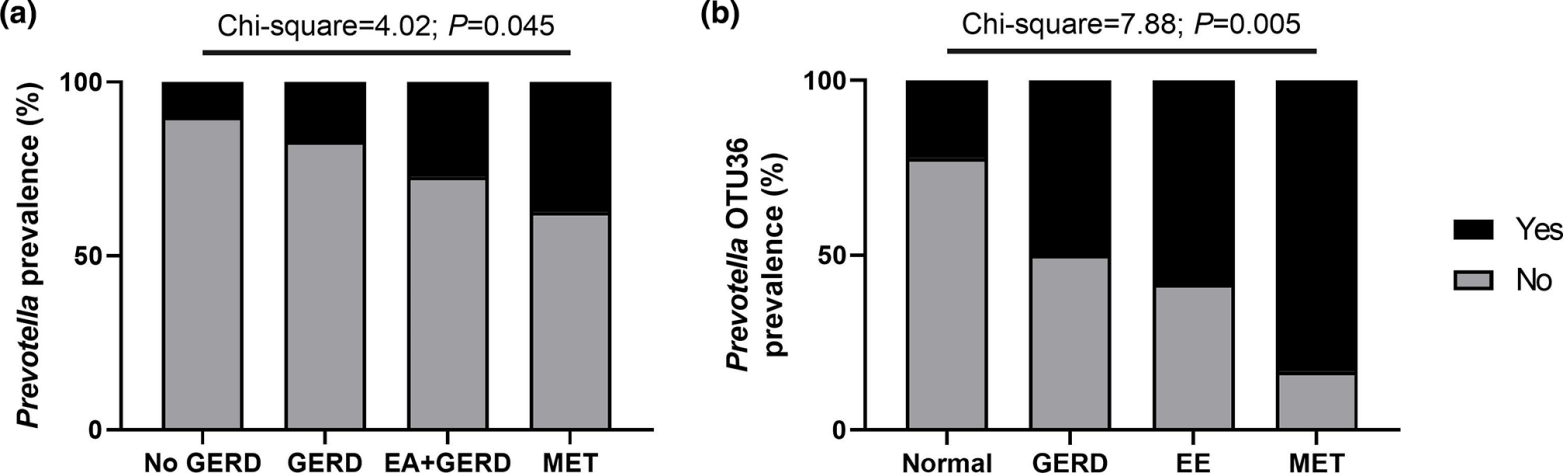
Prevalence of *Prevotella* in the oesophageal microbiota. (a) Prevalence of *Prevotella* across samples collected from the retrospective cohort with no gastro-oesophageal reflux disease (GERD), with GERD, with GERD and oesophageal atresia (EA), or metaplasia (MET). (b) Prevalence of *Prevotella* OTU36 (with similarity to *P. melaninogenica *) across samples collected from the prospective cohort with a normal oesophagus, with gastro-oesophageal reflux disease (GERD), with eosinophilic oesophagitis (EE), or metaplasia (MET). Statistical analyses were performed using chi-square for trend.

To validate the higher levels of taxa classified to *Prevotella* in paediatric oesophageal metaplasia, 100 patients were selected for retrieval of their FFPE oesophageal biopsies. Exclusion criteria were diagnosis of EE and no antibiotic intake. Ninety-six samples were retrieved and sectioned for blinded histological assessment for metaplasia and concurrent DNA extraction and 16S rRNA gene amplicon sequencing (Fig. S5a). Gastric or intestinal metaplasia (Fig. S5b) was detected in 8/96 (8.3 %; 75 % gastric) samples by a blinded pathologist. Microbiota profiling revealed extensive environmental contamination, precluding any assessment of microbiota diversity metrics. This indicated that FFPE samples were not useful surrogates in samples with low-abundance microbiotas. Instead, count tables were analysed for prevalence of *Prevotella* (i.e. any reads classified to the genus *Prevotella *), the genus taxonomic threshold selected to account for environmental contamination, and 17/96 (17.7 %) samples were positive. Importantly, none of the *Prevotella* OTUs were detected in the negative controls. Samples were then stratified post-hoc according to the presence of GERD, and EA +GERD, or metaplasia (MET), where a significant increasing trend (χ^2^=4.02, *P*=0.045; chi-square for trend) in *Prevotella* prevalence was observed (Fig. 5a).

Given this, the prospective cohort was re-examined for the prevalence of *Prevotella* OTU36 (as opposed to relative abundance); 46.3 % (25/54) of prospective patients were positive for *Prevotella* OTU36 in their oesophagus. Patients were then stratified according to results of their histology (i.e. normal, GERD, EE and MET), and a significant increase in positivity was identified with disease (Fig. 5b); 22.2 % (4/18) of patients with a normal oesophagus, 50 % (9/18) of patients with GERD, 58.3 % (7/12) of patients with EE and 83.3 %(5/6) of patients with MET were positive for *Prevotella* OTU36 (χ^2^=7.88, *P*=0.005; chi-square for trend).

### *P. melaninogenica* from patients with metaplasia have distinct genomic features

**Fig. 6.**
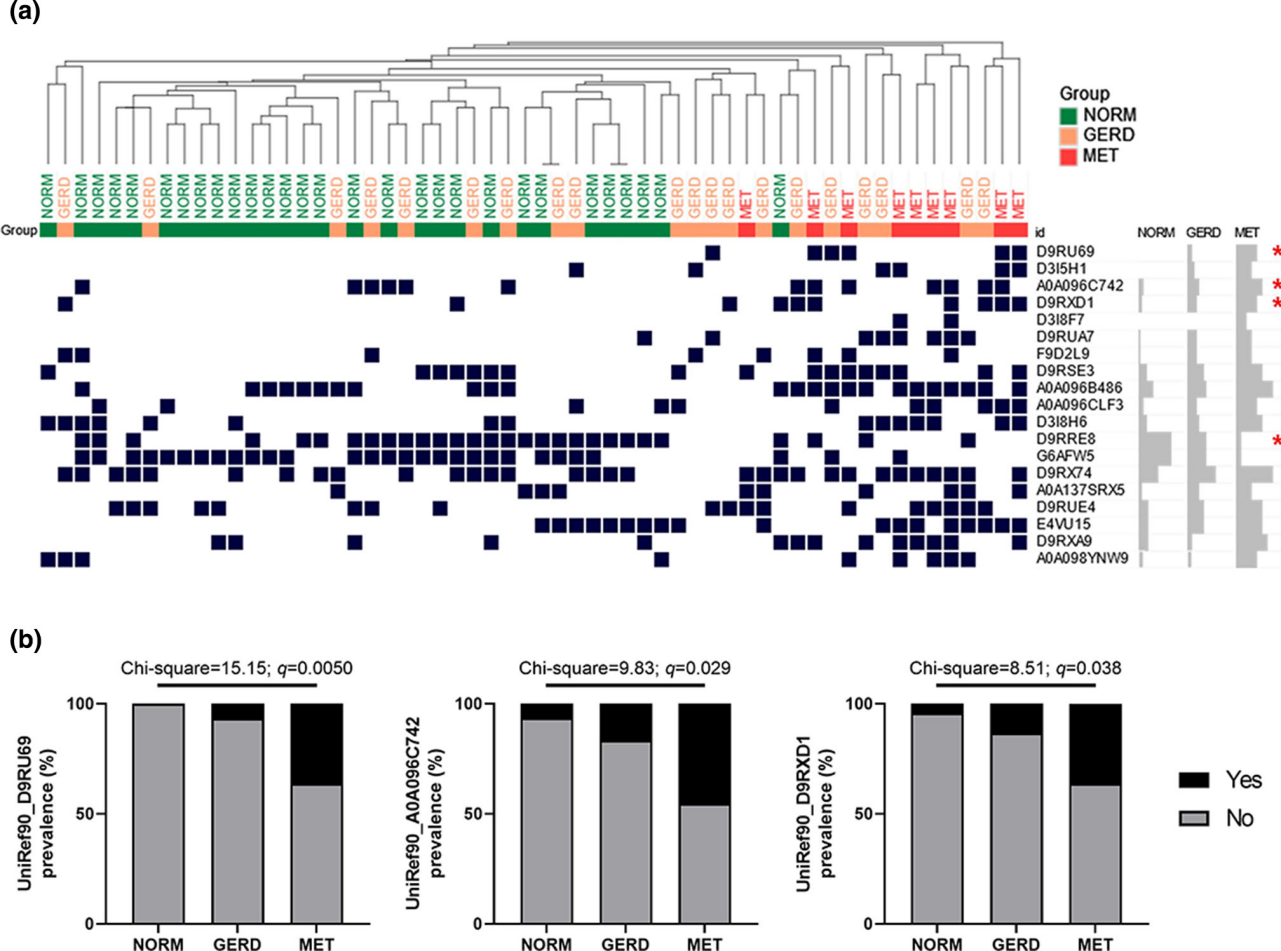
Analysis of *Prevotella melaninogenica* genomes derived from oesophageal metagenomes. (a) Hierarchical clustering analysis (HCA) of Euclidean distances between samples according to 19 differential UniRef features (*q*<0.05) across groups. The *P. melaninogenica* pangenome was used to detect UniRef features using PanPhlAn3 and samples with no detection of *P. melaninogenica* could not be included in the HCA. Statistical differences in the features were calculated using chi-squared for trend test, factoring in samples that were negative for *P. melaninogenica *, followed by correction for FDR. Red asterisks denote selected features. (b) Selected features that increase in prevalence with disease stage. NORM, normal; GERD, gastro-oesophageal reflux disease; MET, metaplasia.

The association of *P. melaninogenica* with oesophageal metaplasia was then further examined in shotgun metagenomics data from oesophageal brushings of an independent adult cohort that was previously published [18]. Shotgun data were screened against the *P. melaninogenica* pangenome and a robust set of genetic features (>2000 features/sample) were detected in 59/88 samples tested (mean±sd: 2692.8±159.4). Feature prevalence was then evaluated using HCA, which suggested those from GERD and MET were not different on the whole (Fig. S6a) but contained a distinct prevalence of certain genetic features when compared to those from NORM (Fig. 6a, Table S7). Analysis of gene ontology indicated an enrichment of membrane proteins (*n*=7) within the 19 features that significantly discriminate NORM, GERD and MET samples (Table S7). The top three features enriched in disease, with at least 30 % prevalence in MET, included a secreted protein with unknown function, a protein with a maintenance of lipid asymmetry domain (MlaD) and another protein of unknown function (Fig. 6b, Fig. S6c). The most discriminatory feature enriched in reads from normal samples was a membrane protein with a TonB_C domain (Fig. S6b,c). This indicated that in addition to being more prevalent/abundant in metaplasia, *P. melaninogenica* genomes from metaplastic samples appear to have distinct features.

## Discussion

We show that the oesophageal microbial community in children is distinct from either the oral or gastric fluid microbiotas, and that all three upper gastrointestinal microbiotas are altered with age and made less compositionally distant intra-patient with GERD. We found that an increased prevalence and abundance of a *Prevotella* taxon with similarity to *P. melaninogenica* is associated with oesophageal metaplasia. Analysis of shotgun metagenomics data against a *P. melaninogenica* pangenome indicated that strains from patients with metaplasia had distinct genetic features that could cluster them separately from strains found in normal oesophagi. Further, while we did not observe an association between PPI use and either the oral or oesophageal microbiotas, these medications were associated with gastric fluid bacterial richness and composition, which in turn were correlated with levels of inflammation.

Profiling of matched oral swabs, oesophageal biopsies and gastric fluid samples from paediatric patients identified significant differences in bacterial alpha and beta diversity across these microbiotas. However, source tracking did suggest that the bacterial component of the oesophageal microbiota, while different in global composition to oral component, originated from the oral cavity. Similar results were reported by our group in adults [4], emphasizing that the bacterial component of the oesophageal microbiota is distinct and not simply a reflection of salivary passage. One variable that was consistently associated with the composition of all three microbiotas was age, suggesting that the upper gastrointestinal environment may be changing dynamically with age. An association between age and the oesophageal microbiota has been previously shown by our group in adults [18]. More importantly, however, these findings may reflect what has recently been reported for the intestinal microbiota, in that it may take longer than 3 years to mature to an adult-like microbiota [22]. In addition to this, the analysis suggested that GERD resulted in these three uppergastrointestinal microbiotas becoming less distant within each patient, a finding that is biologically plausible given the increased exposure to gastric content.

Enrichment of *Prevotella* in oesophageal metaplasia has been reported in adults [23, 24], with Yang *et al*. proposing that a second oesophageal community type dominated by *Prevotella* characterizes metaplasia [24]. Our results from two paediatric cohorts and one adult cohort identify an association between *Prevotella* and oesophageal metaplasia. We observed increased prevalence and abundance across cohorts. However, our findings are novel in that they point to *P. melaninogenica* as the species of interest. This bacterium has been found to induce lung fibrosis in mice through a mechanism driven by induction of IL-17R signalling by bacterial outer-membrane vesicles [25]. Further, the same bacterial strain (ATCC 25845) produces a unique immunoglobulin A protease [26], that could potentially impede mucosal protection against other pathobionts also linked to oesophageal metaplasia [4]. While it is plausible that the change from a squamous to an intestinal-like phenotype or an increase in inflammation could attract this taxon to the oesophagus, the step-wise increase observed across disease stages would argue against the former, with the latter requiring further work to clarify.

We identified discrete genetic features, several of which were membrane proteins, that were differentially prevalent in *P. melaninogenica* from samples with and without metaplasia. This would underscore the need to examine strain-level differences in the microbiota when assigning disease relevance. One of the features was found to contain a region with homology to the maintenance of lipid asymmetry D (MlaD) domain. The MlaD protein is a component of the Mla ABC transport system that traffics phospholipids between the outer and inner membranes of Gram-negative bacteria, and absence of this system leads to accumulation of phospholipids at the outer membrane [27]. The activity has been linked to bacterial antibiotic resistance [28], and the related mammalian cell entry (MCE) proteins in *Mycobacterium tuberculosis* are associated with bacterial utilization of host cholesterol [29]. Future studies should examine if this system provides increased fitness for *P. melaninogenica* or if it is linked to outer membrane vesicle formation, and if this relates to exposure to refluxate and development of metaplasia.

PPI use has been shown to affect the composition of the gastric and intestinal microbiotas [30, 31]. In our cohort, we did observe an effect of PPI use in gastric fluid but not in the oral or oesophageal microbiotas. A similar lack of effect of PPIs on the adult oesophageal microbiota has been reported by us [18]. It is possible that the effect of PPIs is limited to microbial communities that would be directly affected by decreased acid (gastric) or affected by increased microbial passage due to lower acid levels (intestinal). It is also plausible that their effect in the oral and oesophageal environments is weaker and requires larger cohorts to detect; however, this needs to be independently corroborated. We observed an increase in bacterial richness and oral bacterial contribution in the gastric fluid with PPI use, but in addition to this, we found that the changes to the gastric fluid microbiota were associated with increased levels of several inflammatory molecules, indicating that PPI use enables more oral bacterial species to survive the environment, and they in turn could trigger inflammation. The gastrointestinal consequences of PPI use in children requires further investigation.

Our study has several limitations. One limitation is that the number of paediatric patients with metaplasia in our cohort is low and combines gastric and intestinal metaplasia. This is due to the fact that paediatric oesophageal metaplasia is not common. Further, we performed in-depth comparisons of shotgun metagenomics data from adult oesophageal brushings enriched for microbial reads. Future studies can utilize newer strategies to deplete the excess human DNA contamination within oesophageal biopsies to enable shotgun microbial sequencing in samples rich in host DNA.

In conclusion, our results identify a taxon with similarity to *P. melaninogenica* as well as *P. melaninogenica* membrane synthesis proteins to be associated with oesophageal metaplasia. Animal models of oesophageal colonization can clarify whether these species can induce disease or create an environment conducive to disease development, or are simply attracted to the changing environment in disease.

## Supplementary Data

Supplementary material 1

Supplementary material 2
