# Supplementary material for: Changes to the upper gastrointestinal microbiotas of children with reflux oesophagitis and oesophageal metaplasia
Source: Microb Genom. 2022 Sep 15;8(9):mgen000870. doi: 10.1099/mgen.0.000870 (PMC9676027; doi:10.1099/mgen.0.000870)

Figure S1

Quality checks of the data sets. A: Rarefaction curves for the bacterial OTU count data derived from the prospective cohort. Data shows saturation of sequencing across the different sample types. **B:** Bacterial composition of the two negative controls (NEG) relative to the oral (O), esophageal (E) and gastric (G) samples from the prospective cohort. Ordination is a principal coordinate analysis of a Bray-Curtis resemblance matrix derived from square-root transformed relative abundances. **C:** Number of OTUs detected in the negative controls and across the other sample types. **D:** Number of OTUs within the samples that remained after removal of the OTUs that were classified as contaminants due to enrichment in the negative controls. **E:** Bacterial composition of the six negative controls and two sample controls relative to esophageal formalin-fixed parafilm embedded (FFPE) samples from the validation cohort. Ordination is a principal coordinate analysis of a Bray-Curtis resemblance matrix derived from square-root transformed relative abundances. **F:** Number of OTUs detected in the negative controls, sample controls and esophageal samples.

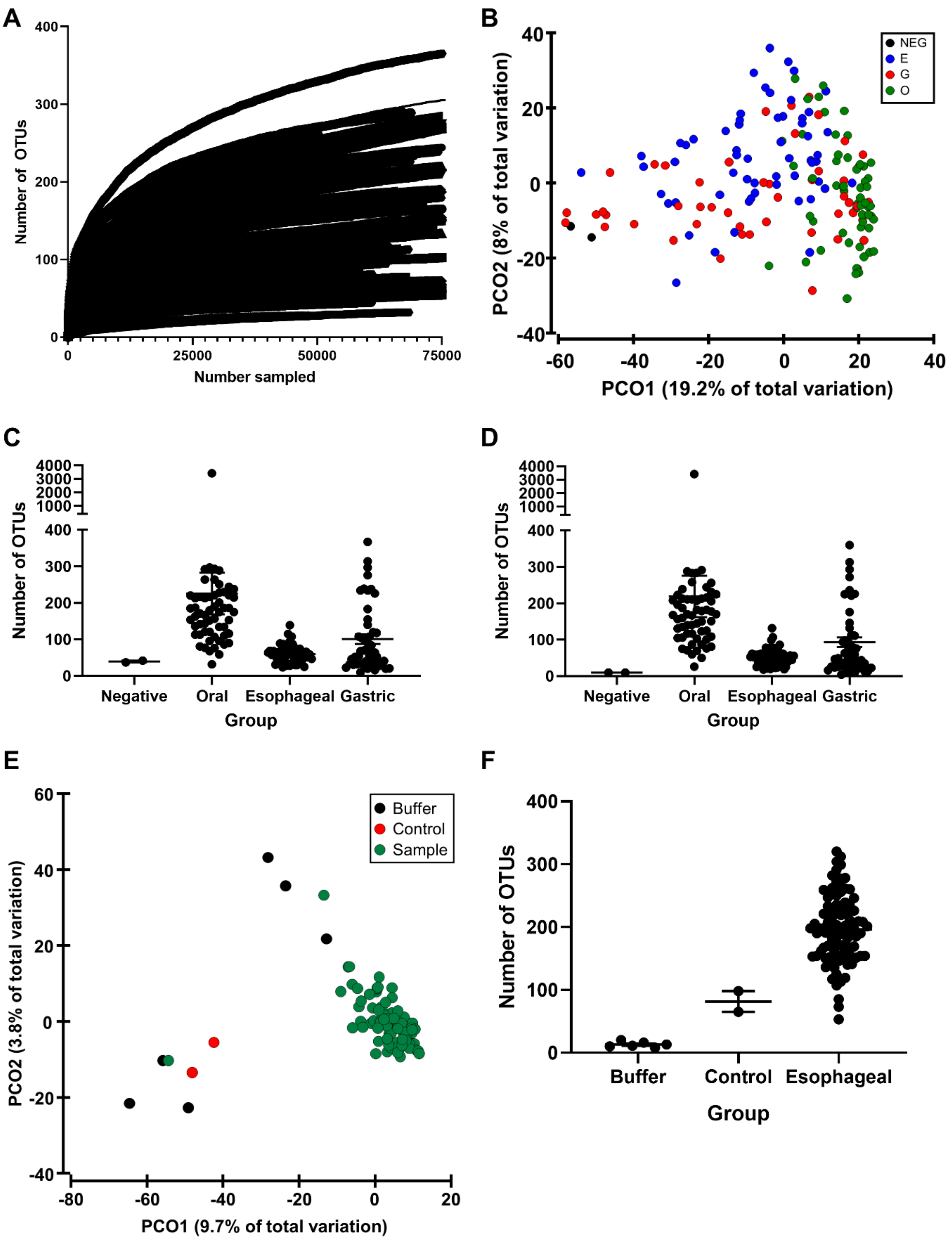
A
Number of OTUs
Number sampled
B
NEG
E
G
O
PCO2 (8% of total variation)
PCO1 (19.2% of total variation)
C
Number of OTUs
Negative
Oral
Esophageal
Gastric
Group
D
Number of OTUs
Negative
Oral
Esophageal
Gastric
Group
E
Buffer
Control
Sample
PCO2 (3.8% of total variation)
PCO1 (9.7% of total variation)
F
Number of OTUs
Buffer
Control
Esophageal
Group

Figure S2

Upper gastrointestinal tract microbiota diversity in a prospective pediatric cohort. A: Bacterial species richness (Main test: $H=63.55$, $p<0.0001$, $n=164$) and evenness (Main test: $H=34.55$, $p<0.0001$, $n=164$) across oral (O), esophageal (E) and gastric fluid (G) samples. Data from one oral sample was considered an outlier (>3400 bacterial OTUs) and removed. **B:** Fungal species richness (Main test: $H=13.37$, $p=0.0013$, $n=162$) and evenness (Main test: $H=0.408$, $p=0.82$, $n=162$) across oral, esophageal and gastric fluid samples. All alpha diversity measures were compared using a Kruskal-Wallis test with a Dunn's multiple comparison test. **C:** Principal coordinate analysis of fungal beta diversity across sample type (oral (O): green; esophageal (E): blue; gastric (G): red). The ordination was generated from a Bray-Curtis resemblance matrix on square-root transformed fungal relative abundances. Groups were compared using ANOSIM.

*p<0.05, **p<0.01, ***p<0.001, ****p<0.0001.

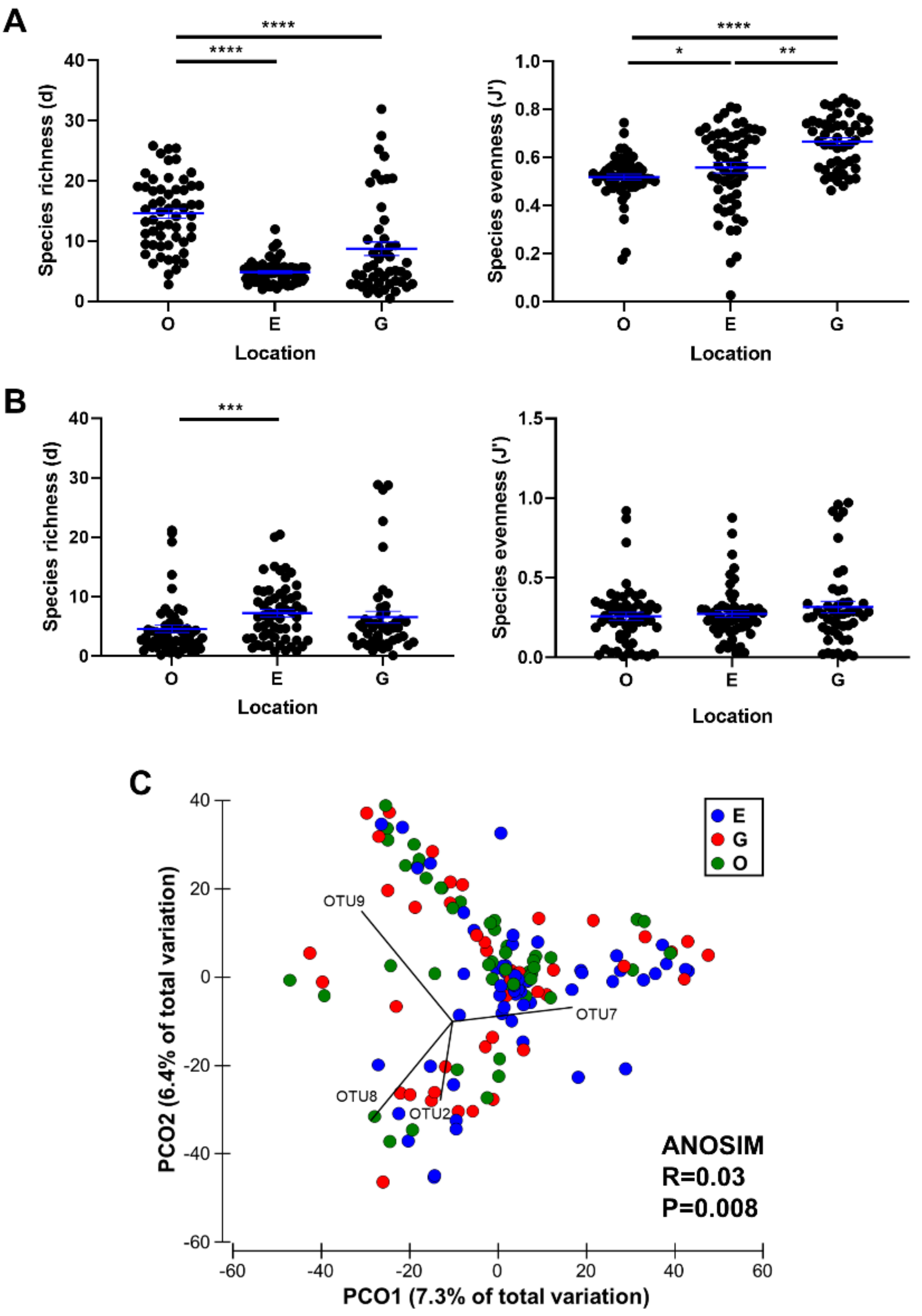
A
****
****
Species richness (d)
40
30
20
10
0
O
E
G
Location
****
*
**
Species evenness (J')
1.0
0.8
0.6
0.4
0.2
0.0
O
E
G
Location
B
***
Species richness (d)
40
30
20
10
0
O
E
G
Location
Species evenness (J')
1.5
1.0
0.5
0.0
O
E
G
Location
C
E
G
O
OTU9
OTU7
OTU8
OTU2
ANOSIM
R=0.03
P=0.008
PCO2 (6.4% of total variation)
40
20
0
-20
-40
-60
-60
-40
-20
0
20
40
60
PCO1 (7.3% of total variation)

Figure S3

Association of oral and esophageal microbiotas with clinical variables. A: Multiple partial correlations between clinical variables and ordination axes. Correlations were generated through constrained ordination using distance-based redundancy analysis (dbRDA). dbRDA was applied on a Bray-Curtis resemblance matrix from square-root transformed bacterial relative abundances. **B:** Constrained ordination of axes 1 and 2 showing differences between oral (O) and esophageal (E) microbiotas.

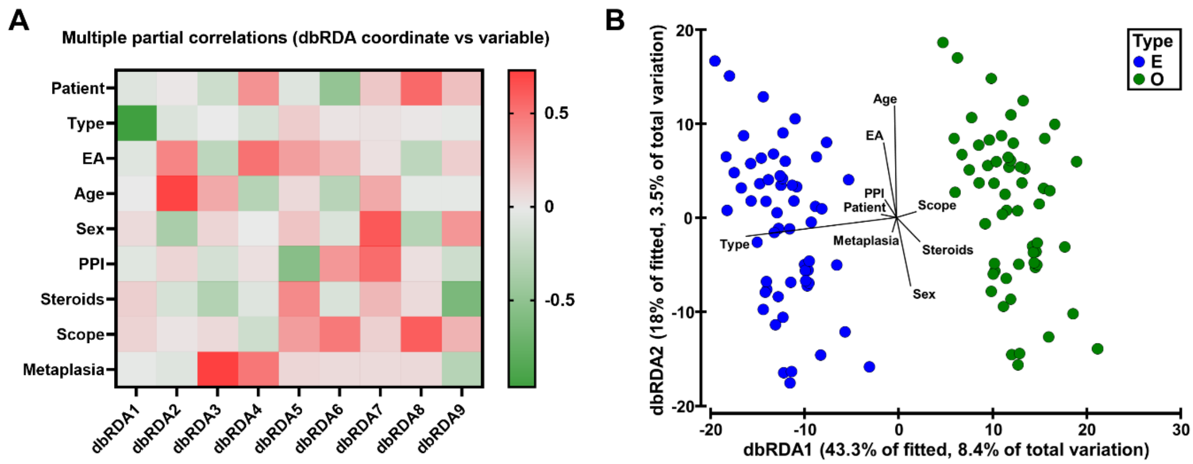

Figure S4

Association of gastric fluid microbiota with clinical variables. Multiple partial correlations between clinical variables and ordination axes. Correlations were generated through constrained ordination using distance-based redundancy analysis (dbRDA). dbRDA was applied on a Bray-Curtis resemblance matrix from square-root transformed bacterial relative abundances. Gradient reflects the correlation values.

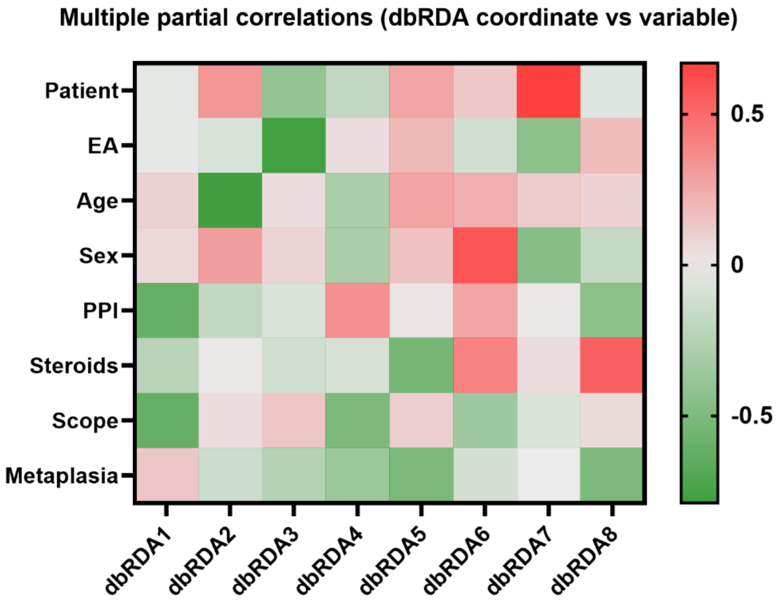

Figure S5

Esophageal microbiota from a retrospective pediatric cohort. A: Summary of patient recruitment and sample processing. Image was created with BioRender.com. **B:** Representative samples of hematoxylin and eosin stained endoscopic esophageal biopsies showing histological features of normal stratified squamous epithelium (Normal, left), pseudostratified columnar cardia or junctional-type metaplasia (MET) with antral-like glands (red rectangle), small areas of gastric foveolar epithelium (blue rectangle) and the absence of goblet cells (MET, right). Imaged at 400X magnification.

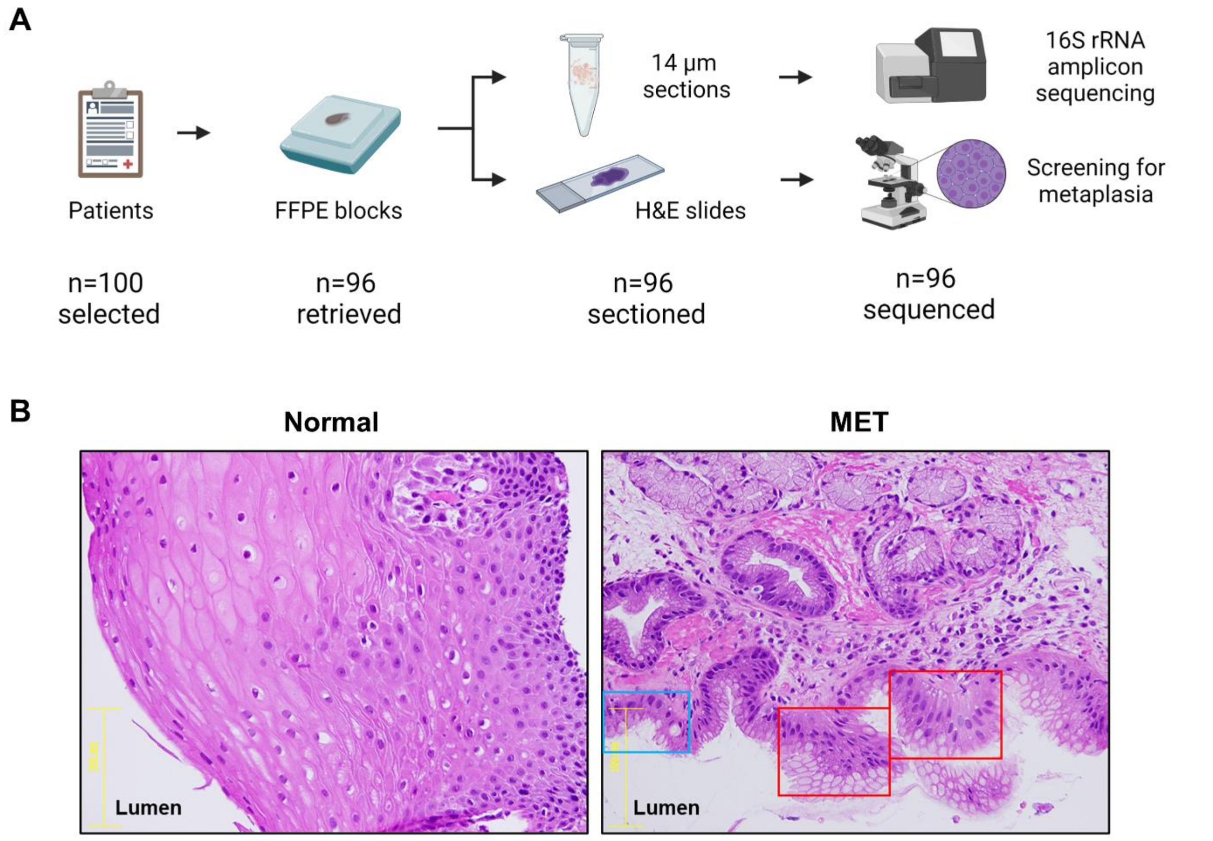

Figure S6

Analysis of *Prevotella melaninogenica* genomes derived from esophageal metagenomes. A: Hierarchical clustering analysis (HCA) of Euclidean distances between samples according to all detected *P. melaninogenica* UniRef features. Samples with no detection of *P. melaninogenica* could not be included. The *P. melaninogenica* pangenome was used to detect UniRef features using PanPhlAn3. **B:** Selected feature that decreases in prevalence with disease stage. **C:** Protein domain analysis of selected differential features using InterPro.

NORM: normal, GERD: gastro-esophageal reflux disease, MET: metaplasia.

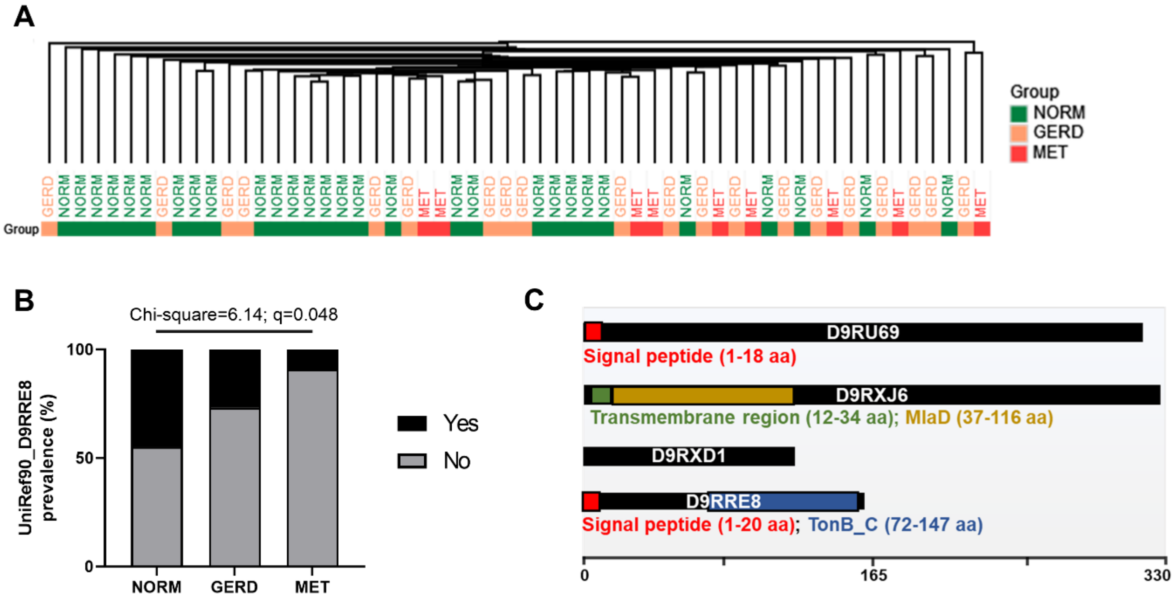

Supplement: Supplementary material 1 [file mgen-8-870-s001.pdf]
